# Use of Colonoscopy and Flexible Sigmoidoscopy Among African Americans and Whites in a Low-Income Population

**Published:** 2007-12-15

**Authors:** Neeraja B Peterson, Harvey J Murff, Jay H Fowke, Yong Cui, Margaret Hargreaves, Lisa B Signorello, William J Blot

**Affiliations:** Department of Medicine, Vanderbilt University Medical Center, Vanderbilt-Ingram Cancer Center, Nashville, Tennessee; Department of Medicine, Vanderbilt University Medical Center, Department of Veteran Affairs, Nashville, Tennessee; Department of Medicine, Vanderbilt University Medical Center, Vanderbilt-Ingram Cancer Center, Nashville, Tennessee; Meharry Medical College, Nashville, Tennessee; Meharry Medical College, Nashville, Tennessee; Department of Medicine, Vanderbilt University Medical Center, Vanderbilt-Ingram Cancer Center, Nashville, Tennessee, International Epidemiology Institute, Rockville, Maryland; Department of Medicine, Vanderbilt University Medical Center, Vanderbilt-Ingram Cancer Center, Nashville, Tennessee, International Epidemiology Institute, Rockville, Maryland

## To the Editor:

Colorectal cancer is the third most common incident cancer and the second most common cause of cancer-related death in the United States. The disease is largely preventable with screening. In the United States, colorectal cancer mortality is higher among African Americans than among whites, possibly because of inequalities in the delivery of screening, diagnostic, and therapeutic regimens (1,2). To elucidate the role of race in the use of two recommended screening tests for colorectal cancer (i.e., colonoscopy and flexible sigmoidoscopy), we compared data on test use among African Americans and white participants in the Southern Community Cohort Study (SCCS).

The SCCS is a large-scale prospective cohort study of cancer (3). We analyzed baseline data collected from participants at enrollment during 2002 through 2006 from 48 community health centers in a 12-state region in the southeastern United States. Sixty-three percent of the participants reported annual household incomes of less than $15,000, and an additional 21% reported annual household incomes of less than $25,000. Questions included "Have you ever had a sigmoidoscopy?" and "Have you ever had a colonoscopy?" If respondents had not had either of the two tests within the recommended time frame or had never had either test, they were asked to indicate a reason using a list we provided. We did not elicit information about screening using fecal occult blood test or barium enema.

Men and women aged 50 years or older at enrollment were eligible for analysis (n = 25,786). We excluded people who were not African American or white (n = 1370) and people who reported a prior diagnosis of colon or rectal cancer (n = 134), resulting in 24,282 subjects for analysis. We defined outcome variables as 1) having ever had testing with a sigmoidoscopy or colonoscopy or 2) having had recommended testing, which is defined by current guidelines (4) as a sigmoidoscopy in the previous 5 years or a colonoscopy in the previous 10 years.

We used chi-square tests with *P* values to compare participants' demographic characteristics (age, race, and sex) and socioeconomic (SES) indexes (annual household income, education, and marital and health insurance status) across categories of test-use status. We used multivariable logistic regression to calculate odds ratios (ORs) and 95% confidence intervals (CIs) summarizing the association between test-use prevalence and race, stratified by sex, after adjustment for demographic and SES variables. We used generalized estimating equations to fit the model to account for the possibility that data were correlated from participants recruited within each community health center.

African American respondents were younger, were less likely to have completed high school, and reported slightly lower household incomes than white respondents. Among all respondents, 38.2% reported having a sigmoidoscopy or colonoscopy, and 34.8% reported having the recommended testing. Having any type of testing was positively associated with increasing age, higher household income, higher education, having been married, having private or public health insurance, and having had a medical visit within the previous year.

Among African Americans, only 30.6% of men and 38.2% of women reported ever having had a sigmoidoscopy or colonoscopy, compared with 38.8% of white men (adjusted OR, 0.93; 95% CI, 0.83–1.03) and 47.4% of white women (adjusted OR, 0.80; 95% CI, 0.72–0.88)(Table 1). When we examined prevalence by type of test, however, we found that the reduced use of endoscopy was entirely accounted for by the reduced use of colonoscopy. African American men and women were similarly likely to have ever had a sigmoidoscopy. The deficit among African Americans in the number who had ever had a colonoscopy was seen in both sexes, but was particularly marked among women (men, adjusted OR, 0.89; 95% CI, 0.80–0.98; women, adjusted OR, 0.70; 95% CI, 0.65–0.76). For both sigmoidoscopy and colonoscopy among African American men and women, the ORs associated with recommended testing were higher (i.e., less of a deficit) than the ORs associated with ever having had the tests. African Americans were significantly more likely than whites to have had a sigmoidoscopy at recommended intervals.

When participants who had not had either recommended test were asked to indicate why, most reported their doctor had not recommended the test (Table 2). The next most common reason was cost.

The limitations of our study included not distinguishing between colonoscopies and sigmoidoscopies performed for screening versus diagnostic purposes, not asking about fecal occult blood tests or barium enemas, and relying on self-reported data.

In summary, we found lower use of colonoscopy among African Americans than among whites. Although future studies are needed to confirm these findings, we suggest that the lower use of colonoscopy may contribute to the higher rates of colorectal cancer mortality among African Americans.

## Figures and Tables

**Table 1:** Prevalence of Endoscopic Testing for Colorectal Cancer Among African American and White Men and Women, Southern Community Cohort Study, 2002—2006

Test	African American Men (n = 6945)^a^ No. (%)	White Men (n = 1938)^a^ No. (%)	OR, Unadjusted^b^ (95% CI)	OR, Adjusted^b^,^c^ (95% CI)
**Sigmoidoscopy**				
Ever had test	1148 (16.8)	331 (17.3)	0.96 (0.79-1.16)	1.12 (0.95-1.33)
Recommended testing^d^	970 (14.2)	237 (12.4)	1.18 (0.95-1.48)	1.39 (1.14-1.70)
**Colonoscopy**				
Ever had test	1433 (21.0)	581 (30.4)	0.77 (0.68-0.86)	0.89 (0.80-0.98)
Recommended testing^d^	1352 (19.9)	536 (28.1)	0.81 (0.72-0.91)	0.94 (0.84-1.06)
**Either test**				
Ever had test(s)	2092 (30.6)	742 (38.8)	0.79 (0.70-0.89)	0.93 (0.83-1.03)
Recommended testing^d^	1907 (28.1)	650 (34.2)	0.88 (0.77-1.01)	1.04 (0.91-1.19)

**Test**	**African American Women (n = 11,163)^a^ No. (%)**	**White Women (n = 4236)^a^ No. (%)**	**OR, Unadjusted^b^ (95% CI)**	**OR, Adjusted^b^,^c^ (95% CI)**

**Sigmoidoscopy**				
Ever had test	1979 (18.0)	790 (18.8)	0.98 (0.85-1.14)	1.00 (0.86-1.16)
Recommended testing^d^	1573 (14.4)	517 (12.4)	1.24 (1.07-1.44)	1.24 (1.07-1.45)
**Colonoscopy**				
Ever had test	3106 (28.3)	1701 (40.5)	0.68 (0.62-0.75)	0.70 (0.65-0.76)
Recommended testing^d^	2943 (26.9)	1556 (37.2)	0.73 (0.67-0.80)	0.75 (0.70-0.81)
**Either test**				
Ever had test(s)	4201 (38.2)	1990 (47.4)	0.78 (0.71-0.87)	0.80 (0.72-0.88)
Recommended testing^d^	3787 (34.8)	1726 (41.3)	0.87 (0.79-0.96)	0.89 (0.82-0.98)

OR indicates odds ratio; CI, confidence interval.

a Approximately 2% of outcome data in each test category was missing from analysis.

b Referent group is white study participants of same sex.

c Adjusted for age, education, income, and marital and insurance status.

d Recommended testing refers to having a sigmoidoscopy within the last 5 years or having a colonoscopy within the last 10 years.

**Table 2:** Reasons Given by African American and White Participants for Not Having Recommended Endoscopic Testing for Colorectal Cancer, Southern Community Cohort Study, 2002—2006^a^

Reason	African American (n = 11,994) No. (%)	White (n = 3706) No. (%)	***P* value^b^**
Doctor did not recommend test	7698 (64.2)	2245 (60.6)	<.001
Forgot to do it	215 (1.8)	59 (1.6)	.41
Have fear of finding cancer	314 (2.6)	90 (2.4)	.52
Put it off or too busy	450 (3.8)	202 (5.5)	<.001
Embarrassment	109 (0.9)	77 (2.1)	<.001
Cost	881 (7.4)	525 (14.2)	<.001
May experience pain or discomfort during the test	413 (3.5)	209 (5.6)	<.001
None of the above	2528 (21.1)	682 (18.4)	<.001

a Among participants who reported not having a sigmoidoscopy within the last 5 years or a colonoscopy within the last 10 years. Participants were permitted to select more than one reason.

b *P* values were determined by chi-square tests.

## References

[1] Mayberry RM, Coates RJ, Hill HA, Click LA, Chen VW, Austin DF (1995). Determinants of black/white differences in colon cancer survival. J Natl Cancer Inst.

[2] Ries LA, Wingo PA, Miller DS, Howe HL, Weir HK, Rosenberg HM (2000). The annual report to the nation on the status of cancer, 1973-1997, with a special section on colorectal cancer. Cancer.

[3] Signorello LB, Hargreaves MK, Steinwandel MD, Zheng W, Cai Q, Schlundt DG (2005). Southern community cohort study: establishing a cohort to investigate health disparities. J Natl Med Assoc.

[4] (2002). U.S. Preventive Services Task Force. Screening for colorectal cancer: recommendation and rationale. Ann Intern Med.

